# Patients with high levels of circulating endothelial progenitor cells (EPC) following at least three months of anticoagulation for unprovoked venous thromboembolism (VTE) are at low risk of recurrent VTE—Results from the ExACT randomised controlled trial.

**DOI:** 10.1016/j.eclinm.2019.11.011

**Published:** 2019-11-27

**Authors:** Charlotte Bradbury, Tracey Buckley, Yong Zhong Sun, Peter Rose, David Fitzmaurice

**Affiliations:** a School of Cellular and Molecular Medicine, University of Bristol, United Kingdom; b University Hospitals Bristol, United Kingdom; c University Hospitals Birmingham, United Kingdom; d University of Birmingham, United Kingdom; e University Hospitals Coventry and Warwickshire, United Kingdom; f University of Warwick, United Kingdom

**Keywords:** Anticoagulation, d-dimers, Endothelial progenitor cells, Recurrence, Venous thrombosis, VTE

## Abstract

**Background:**

There is clinical need for a laboratory biomarker to identify patients who, following an unprovoked venous thrombosis (VTE), are at low VTE recurrence risk and can discontinue anticoagulation after a limited treatment duration (3–6 m). This secondary analysis of the ExACT study aimed to evaluate whether quantitation of peripheral blood endothelial progenitor cells (EPCs) could improve prediction of VTE recurrence risk.

**Methods:**

The ExACT study was a non-blinded, multicentre RCT comparing extended vs discontinued anticoagulation following a first unprovoked VTE. Adult patients were eligible if they had completed ≥3 months anticoagulation and remained anticoagulated. The primary outcome was time to first recurrent VTE from randomisation. Blood samples were taken at baseline and results correlated with clinical outcome over 2 years follow up. (Trial registration: ISRCTN:73819751 and EUDRACT:2101-022119-20)

**Findings:**

281 patients were recruited, randomised (between July 2011 and February 2015) and followed up for 24 months (Male:Female 2:1, mean age 63). Of these, 273 patients were included in the final analysis. Blood samples were received at baseline for Full Blood Count(*n* = 216), d-dimers(*n* = 205) and endothelial progenitor cell (EPC) quantitation by flow cytometry(*n* = 193). VTE recurrence was lower in the extended vs discontinued anticoagulation arms (5% vs 23%, HR 0.20(95%CI:0.09–0.46,*p* < 0.001)). Level of EPCs were lower in patients who later developed VTE recurrence (43.41 ± 7.69 cells/ml vs 87.1 ± 7.15 cells/ml, *p* = 0.02). Survival free from VTE recurrence was significantly improved in patients with EPCs ≥ 100 cells/ml vs EPCs < 100 cells/ml (HR 0.10(95%CI:0.01–0.75,*p* = 0.025)).

**Interpretation:**

If confirmed, EPC quantitation may represent a novel biomarker that identifies patients at low VTE recurrence risk who are suitable for limited duration anticoagulation.

**Research in context**

**Evidence before this study**

Guidelines recommend consideration of long-term anticoagulation following an unprovoked VTE if risk of VTE recurrence outweighs risk of anticoagulant related bleeding. However, it is increasingly challenging to identify patients who can safely stop after limited duration therapy (3–6 months). d-dimer testing is included in many risk scores but most of these test D-dimers after discontinuation of anticoagulation for one month which is logistically complex and potentially harmful risking VTE recurrence whilst awaiting testing. In addition, d-dimers are poor at defining patients at low recurrence risk who can stop anticoagulation.

**Added value of this study**

This study is the first to report that quantification of circulating endothelial progenitor cells (EPCs) may define a group of patients at low risk of VTE recurrence. This relatively straightforward assay can be done on anticoagulated patients overcoming the limitations of d-dimer testing.

**Implications of all the available evidence**

If these findings are confirmed, EPC quantification is expected to influence clinical decisions by defining a group of patients suitable to discontinue anticoagulation after a defined treatment period. The advantages of stopping treatment in those who no longer need it, include reduced bleeding risk, financial and resource cost savings and patient convenience. Consistent with the published literature, these results also support a mechanistic importance of EPCs following VTE with potential for a new avenue of therapeutic strategies.

Alt-text: Unlabelled box

## 1 Introduction

Venous thromboembolism (VTE) is a prevalent and severe disease, affecting approximately 1–2 per 1000 per year with a risk of death, recurrence, long term morbidity and impaired quality of life (QoL). Anticoagulation therapy (AT) is the mainstay of VTE treatment with a minimum of 3 months duration recommended. Beyond 3 months, a decision must be made to determine which patients warrant long term, indefinite duration anticoagulation for secondary prevention of VTE recurrence. This decision needs to balance the risk of recurrence if AT is stopped and the risk of bleeding if AT continues. The most important factor to consider when estimating the VTE recurrence risk is the clinical circumstance of the first event. The risk of recurrence after an unprovoked VTE (no obvious precipitant) is higher than following a provoked VTE with a transient risk factor (such as surgery) [1]. Clinical guidelines recommend consideration of long-term anticoagulation in patients with an unprovoked proximal deep vein thrombosis (DVT) or pulmonary embolism (PE) after weighing up individual additional risk factors for recurrence, bleeding risk and patient preference (NICE CG144 2015, ACCP 2016, BSH 2011) [2,3]. Other factors known to be associated with increased VTE recurrence risk include male sex [4], raised d-dimer after cessation of anticoagulation for 1 month [5,6] and post thrombotic syndrome [7].

Over recent years, direct oral anticoagulants (DOACs) have been tested for the secondary prevention of recurrent VTE (beyond the first 3–6 months of treatment) with lower, prophylactic doses shown to have good efficacy and safety. Apixaban 2.5 mg bd has demonstrated a comparable incidence of major bleeding to placebo [8] and rivaroxaban (10 mg od or 20 mg od) a comparable bleeding risk to aspirin [9]. Therefore, with anticoagulant options that are safer and more convenient than warfarin, it becomes increasingly important to identify a group of patients who can safely stop anticoagulation after an unprovoked VTE. Although d-dimers may help identify a high-risk group, a negative result does not define a low-risk group. For example, male patients with a negative d-dimer were shown to have an ongoing annual risk of recurrence of 7.5% with extended follow up (median of 5 years) [10]. In addition, there are logistical difficulties in the measurement of d-dimers 1 month after stopping anticoagulation and patients may be at risk of a recurrent event while awaiting testing. Therefore, an ideal biomarker would be one that could be tested when patients are still on anticoagulation and the result could define a group at very low risk of recurrence in whom it is reasonable to discontinue anticoagulation after limited duration anticoagulation treatment, for example 3 months.

Circulating endothelial cells (CECs) are recognised as markers of vascular injury [11]. They can be derived from the vascular wall (mature CEC) or from the bone marrow (Endothelial progenitor cells, EPC [12,13]). EPCs are recruited from the bone marrow in response to stimuli such as ischaemia including myocardial infarction, ischaemic stroke, vascular trauma, sickle cell anaemia, vasculitis and pulmonary hypertension. They have proliferative and angiogenic potential and have been implicated in vascular repair, helping restore the integrity of the endothelium [14].

There is growing interest in the prognostic and therapeutic implications of EPCs in arterial disease. Fadini et al. [15] demonstrated that EPCs are significantly reduced in diabetic patients with peripheral arterial disease (PAD) compared to diabetic patients without PAD and in patients with PAD compared to healthy controls. Circulating EPCs are also significantly lower in patients with coronary artery disease and associated with progression of disease angioradiographically [16,17]. Further observational studies have shown that reduced baseline levels of EPCs and CECs are associated with a significant increased risk of many types of cardiovascular events, all-cause death, and onset/progression of microangiopathy [17]. The most predictive phenotypes were CD34+ and CD34+CD133+. The main limitation of these studies is the high heterogeneity in terms of patient characteristics and cell phenotypes. There is a growing interest in the therapeutic role of EPCs to improve cardiovascular outcomes. Several studies have found lipid lowering statins and some types of antihypertensives increase the level of endogenous circulating EPCs which may contribute to their therapeutic benefit in cardiovascular disease [18,19]. In addition, coronary stents have been developed to “capture” EPCs with the aim to improve endothelialisation and prevent stent stenosis [20].

Although there is a growing literature highlighting the importance of EPCs in arterial disease, their role in venous thrombotic disease remains remarkably understudied. However, there are mechanistic reasons to hypothesise that they would have a similar “vascular repair and protect” role in venous thrombotic disease [21] and preliminary data in mice and small observational studies support this hypothesis [22,23]. However, there are no published data on the measurement of EPCs in a large population of patients with VTE and comparison to clinical outcomes and other biomarkers known to be predictive of recurrence risk for example, d-dimer.

The aim of the ExACT study was to investigate the effect of extended treatment with oral anticoagulation for those patients with first unprovoked proximal DVT or PE in terms of recurrent VTE and post thrombotic syndrome (PTS) [24]. Patients with a first unprovoked VTE who had completed a minimum of 3 months of anticoagulation were randomised to either extended or discontinued AT. Blood samples were taken from patients at baseline whilst still on anticoagulation. The main trial findings and point of care d-dimer testing results are published separately [25]. We hypothesised that quantitation of circulating endothelial progenitor cells may be a candidate predictive biomarker of VTE recurrence risk to aid clinical decision making on anticoagulation duration. The quantitation of EPCs and correlation with clinical outcome (VTE recurrence) was pre-specified prior to patient recruitment, but due to the lack of previous data in this clinical context, the EPC level cut off threshold was determined post-hoc.

## 2 Methods

### 2.1 Trial design and participants

ExACT was a non-blinded, multi-centre, two-arm, parallel-group RCT. The full methods for ExACT study have been published [24]. An open label trial design was chosen to be cost effective and pragmatic due to the costs and complexities associated with a warfarin placebo with INR monitoring and dose adjustment. Eligible patients were aged ≥18 years with a first unprovoked proximal DVT or PE who had completed ≥ 3 months anticoagulation (target INR 2–3 for those taking warfarin) and remained anticoagulated. Patients were excluded if they had another indication for long-term anticoagulation (e.g. AF), were at high risk of bleeding (e.g. additional antiplatelet) or very high risk of VTE recurrence (e.g. antiphospholipid syndrome or active cancer) or a life expectancy <5 years.

Ethics permission was granted by Trent Research Ethics Committee; reference 11/H0605/5. The trial is registered **(**ISRCTN:73819751 and EUDRACT:2101-022119-20). Trial oversight was by an independent Data Monitoring Committee (IDMC) and a Trial Steering Committee (TSC).

### 2.2 Recruitment, randomisation and intervention

Patients were identified from 35 UK NHS anticoagulant clinics (25 secondary and 10 primary care sites). Patients who gave informed, written consent were randomised (1:1) to either extended AT for 24 months or discontinued AT. Randomisation was performed within the web-based computerised clinical case report form. The software used random blocks randomisation (block size of 4) stratified by diagnosis (DVT or PE). All participants were asked to attend 6 monthly study follow-up clinic appointments for two years (5 visits in total). During each study follow-up appointment and at the end of the study, participants’ GP records were reviewed for evidence of any thrombotic or haemorrhagic events (including fatalities), whether the participant was taking an anticoagulant, and any hospital admissions. The primary study outcome was the time to first recurrent venous thromboembolism (VTE) between randomisation and 24 months. All potential thrombotic and haemorrhagic events were scrutinised by an Independent Adjudication Committee who were blind to the intervention allocation.

The baseline appointment was carried out prior to cessation of anticoagulation. At the baseline clinic appointment blood tests were performed.

## 3 Blood samples

Peripheral blood was collected from patients at the baseline appointment (all patients were still on anticoagulation). The blood samples were taken by sterile venepuncture into ethylenediaminetetraacetic (EDTA) and citrate containing tubes and transported the same day at room temperature. Samples were processed centrally at the University of Warwick within 24 h of venepuncture for Full Blood Count (FBC), INR, d-dimers and circulating endothelial cells by flow cytometry. FBCs were analysed on the Sysmex XE-2100 and d-dimers on the Sysmex CS-2100, using the innovance d-dimer kit.

**Fig. 1:**
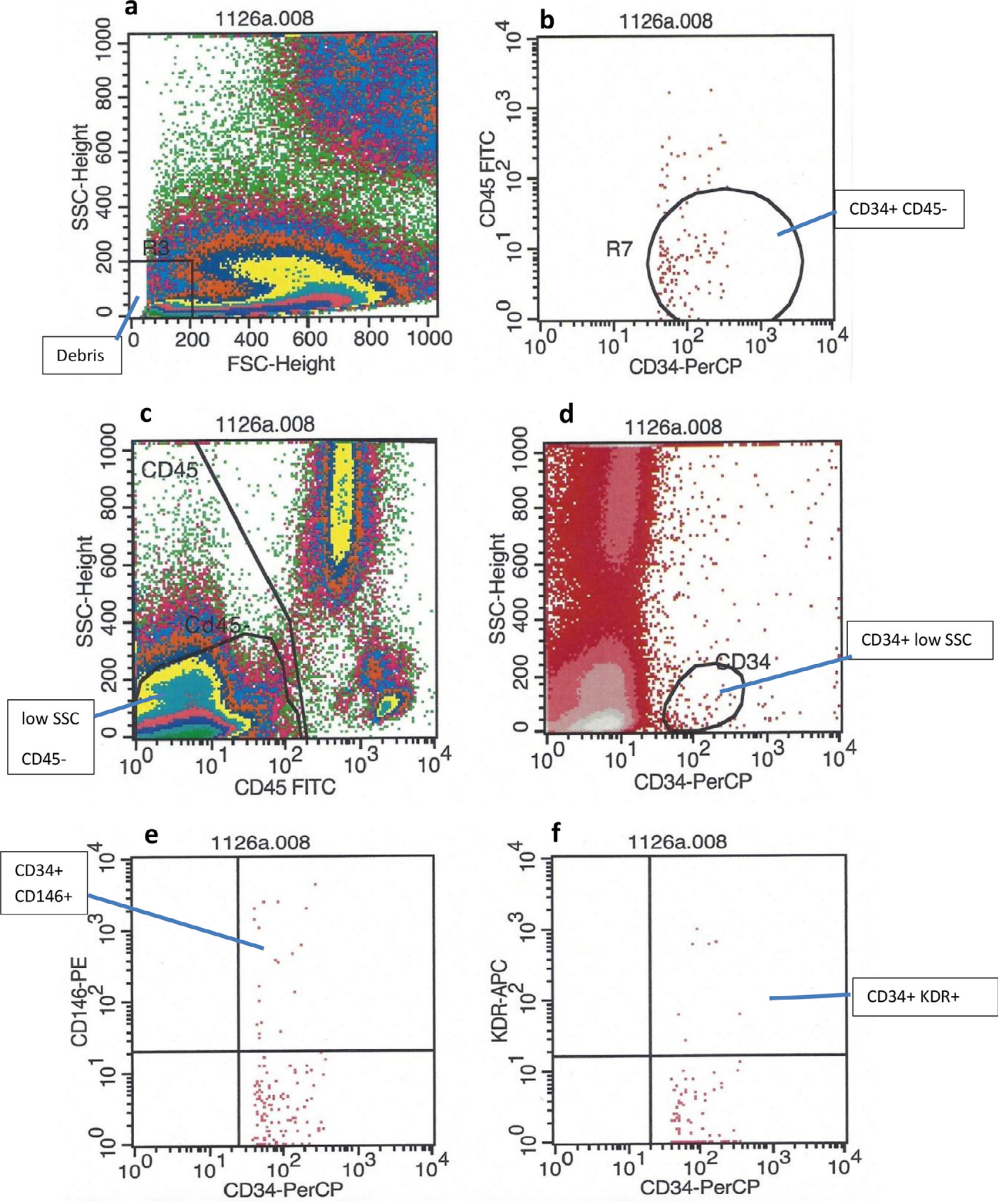
Endothelial progenitor cells (EPCs) were identified by weak CD45 (unlike haematopoetic cells), low side scatter, expression of immaturity (CD34, b and d) and expression of endothelial markers (CD146, VEGFR-2/KDR, e and f). Cells in panels e and f are derived from the low SSC, CD45- gate (panel c).

Samples for flow cytometry were processed in duplicate to ensure reproducibility as the detection of endothelial cells is classed as a “rare event”. In brief, 200 µl of well mixed blood, 10 µl of the antibodies CD45, CD34, CD146, anti-hVEGF R2KDR and 200 µl Isoflow (Beckman Coulter) were added to each of 2 polybrene test tubes. Samples were then incubated in the dark for 30 min before 3 ml of a 1:10 concentration of BD FACS lysing solution (Becton Dickinson) was added to each tube followed by a further incubation in the dark for 10 min. Samples were then centrifuged at 500×g for 5 min and then the supernatant was removed and the pellet resuspended in 3 ml of Isoflow and mixed by gentle inversion. The samples were then centrifuged again 500×g for 5 min, supernatant removed, pellet re-suspended in 0.5 ml of Isoflow and mixed again by gentle inversion. Both tubes were then analysed by flow cytometry (Beckman Coulter Naviosflow cytometer) and data was analysed using Navios System Software FACSDiva software. Endothelial cells were identified by low side scatter, weak CD45 (unlike haematopoetic cells) and expression of endothelial markers (CD146, VEGFR-2/KDR). Endothelial progenitor cells (EPC) also demonstrated at least one marker of immaturity (CD34), whereas mature circulating endothelial cells (mCECs) lacked a marker of immaturity (Fig. 1, Table 1). The number of EPC and mCEC events was divided by the number of CD45 positive events and multiplied by the total white count (from FBC) to give a value × 10^6^/ml. For the number of cells/ml this was multiplied by 10^6^. The laboratory researcher processing and analysing the laboratory samples was blinded to treatment allocation and clinical outcome.

**Table 1:** List of antibodies used in flow cytometry studies.

Antigen	Species	Fluorochrome	Manufacturer
CD45	Cat	FITC	Becton Dickson Ltd
CD146	Cat	PE	Becton Dickson Ltd
CD34	Cat	PC7	Becton Dickson Ltd
h-VEGF R/KDR	Cat	APC	R and D systems Europe

### 3.1 Sample size

The ExACT study was designed to compare 2-year VTE recurrence rates between participants in the extended versus discontinued AT arms, and also to compare these rates for a group of participants with a baseline raised d-dimer [26]. A sample size of 352 (176 per arm) would be sufficient to detect a clinically important difference between the arms with minimum 86% power, two-sided alpha=0.05, assuming recurrence rates between 1.4% and 4.3% for the extended AT arm and 14.2% in the discontinued AT arm [27]. Recruitment was lower than expected and at the TSC request, the power calculation was re-estimated where it was determined that a sample of 270 participants (allowing for 10% loss to follow up) would provide at least 80% power to detect the planned effect sizes.

### 3.2 Analysis

As reported in the main trial paper [25], all primary analyses (primary and secondary outcomes) were performed on an intention to treat basis (ITT).

The number and percentage of participants with at least one recurrent VTE is presented by trial arm. Cox regression analysis was used to compare the time to first recurrent VTE between randomisation arms, censoring for deaths, losses to follow-up and withdrawals of consent to use data. The analysis was adjusted for diagnosis (DVT/PE) at baseline. The proportional hazards assumption was tested by cumulative log hazard plots and including a time by treatment covariate in the analysis.

For this secondary study, statistical analysis was performed using GraphPad Prism® (V6.0 for Windows, GraphPad Software, San Diego California USA), Stata version 12 and SPSS® (IBM 2017). White cell count (WCC), EPCs, mCECs and d-dimer results were expressed as means and standard deviations with comparisons made between groups by two sample t tests. The statistical package SPSS was used to produce Kaplan Meier survival curves to compare the survival free from VTE recurrence between 2 groups of sex (male vs. female), EPC (high vs. low) and d-dimer (positive vs. negative). As there was no previous data on EPC quantitation in this context, the threshold for High versus Low was chosen after the flow cytometry analysis had been completed with the specific aim to define a clinically useful threshold that could identify patients at low risk of VTE recurrence. Cox regression analyses were used and adjusted for treatment allocation to produce adjusted hazard ratios, associated 95% confidence intervals and p-values for VTE recurrences between the comparison groups. In all cases a p-value of less than 0.05 was considered significant.

## 4 Results

### 4.1 Participant recruitment

The first patient was randomised in July 2011 and the last in February 2015. Two hundred and eighty-one patients provided written informed consent to participate (279 on warfarin and 2 on rivaroxaban). All patients who had research blood samples taken were on warfarin.

Patients were randomised (141 to extended anticoagulation and 140 to discontinued anticoagulation) and of these, 273 patients were included in the final analysis of time to first VTE recurrence (median age was 64 years, with a roughly even split between DVT and PE, whilst 67% of participants were male). All 281 trial participants attended visit 1, 273/281 (97%) attended visit 2, 263 (94%) attended visit 3 and 260/281 (93%) visit 4.

Six participants in the discontinued AT group (4 withdrawals, 1 protein S deficiency and 1 antithrombin deficiency) and two in the extended AT group (1 withdrawal and 1 antiphospholipid syndrome) were excluded from the final ITT analysis by post-randomisation pre-defined exclusions [24].

### 4.2 Thrombosis recurrence

Thrombosis recurrence occurred in 14% (*n* = 38 of 273) of patients overall. There was strong evidence of reduced VTE recurrence in the extended anticoagulation vs discontinued anticoagulation arms (5%, *n* = 7 of 139 vs 23%, *n* = 31 of 134, adjusted hazard ratio of 0.20 (95% CI: 0.09–0.46, *p* < 0.001 by Cox regression) (Tables 2 and 3, Fig. 3).

**Table 2:** Adjusted hazard ratios based on Cox regressions, to compare VTE recurrences for extended anticoagulation (AT) versus discontinued AT group, male versus female, positive d-dimer (≥0.5 mg/L) versus negative d-dimer (<0.5 mg/L), EPC high (≥ 100 cells/ml) versus low (<100 cells/ml) and to look at age effect on VTE recurrences.

Baseline characteristics	Recurrent venous thromboembolism (*N* = 38)	No recurrence (*N* = 235)	Adjusted hazard ratio† (95% CI)	P-value
Treatment				
Discontinued AT, N (row%)	31 (23)	103 (77)	0.20	<0.001
Extended AT, N (row%)	7 (5)	132 (95)	(0.09, 0.46)	
Sex				
Female, N (row%)	12 (13)	77 (87)	1.13	0.734
Male, N (row%)	26 (14)	158 (86)	(0.57, 2.24)	
D-dimer				
Negative, N (row%)	19 (12)	141 (88)	1.41±	0.400
Positive, N (row%)	9 (20)	36 (80)		
Missing, N (row%)	10 (15)	58 (85)	(0.63, 3.14)	
EPC€				
Low, N (row%)	23 (17)	115 (83)	0.10‡	0.023
High, N (row%)	1 (2)	54 (98)		
Not done, N (row%)	14 (18)	66 (83)	(0.01, 0.73)	
Age				
Mean (SD)	65.0 (15.1)	62.4 (12.4)	1.01¥	0.347
Median [IQR]	65.3 [53.8, 77.1]	64.2 [54.4, 72.4]		
Range	28.9–92.8	27.9–86.6	(0.99, 1.04)	

† Where appropriate, all hazard ratios were generated based on Cox regression models adjusted for treatment group and baseline diagnosis (DVT/PE), where the baseline diagnosis is the stratification variable for randomisation.

± 68 participants with missing d-dimer were excluded from the Cox regression.

‡ 80 participants whose EPCs were not measured were excluded from the Cox regression.

¥ The hazard ratio is for each unit increase of age.

€ Endothelial progenitor cells.

**Table 3:** Patient and laboratory characteristics according to randomisation group and presence or absence of venous thromboembolism (VTE) recurrence over 2 years follow up. AT= Anticoagulation therapy, EPC= Endothelial progenitor cell quantitation in the peripheral blood.

		All *n* = 273	Sex		Age	EPC *n* = 193		D Dimer *n* = 205	
Group allocation			Male	Female	Median *years*	Low <100 cells/ml	High ≥100 cells/ml	Positive ≥0.5 mg/L	Negative <0.5 mg/L
Discontinued AT	VTE recurrence (n, % of column, discontinued AT)	31 (23.1%)	23 (25.6%)	8 (18.2%)	63	19 (29.7%)	0 (0%)	5 (18.5%)	17 (23.6%)
	No VTE	103	67	36	64	45	25	22	55
	Total	134	90	44	64	64	25	27	72

Extended AT	VTE recurrence (n, % of column, extended AT)	7 (5%)	3 (3.2%)	4 (8.9%)	77	4 (5.4%)	1 (3.3%)	4 (22.2%)	2 (2.2%)
	No VTE	132	91	41	63	70	29	14	86
	Total	139	94	45	63	74	30	18	88

All patients	VTE recurrence (n, % of column, all pts)	38 (13.9%)	26 (14.1%)	12 (13.5%)	64	23 (16.7%)	1 (1.8%)	8 (17.8%)	19 (11.9%)
	No VTE	235	158	77	64	115	54	37	141
	Total	273	184	89	64	138	55	45	160

In this cohort, there was no significant difference in recurrence in men versus women (14%, *n* = 26 of 184 males versus 13%, *n* = 12 of 89 females, adjusted hazard ratio of 1.13 (95% CI: 0.57-2.24, *p* = 0.734)) based on a Cox regression model (Table 2**,** Fig. 3) and there was no difference in age between patients who developed recurrence versus those who didn't (VTE recurrence median 65 years, range 29–93 years, versus no recurrence median 64 years range 28–87 years, *p* = 0.347). The Cox regression analysis showed for each 1 year increase in age, the hazard rate of VTE recurrence increased by 2% but the result is not significant (95% CI: 0.99–1.04, *p* = 0.242).

### 4.3 Endothelial progenitor cell quantitation

**Fig. 2:**
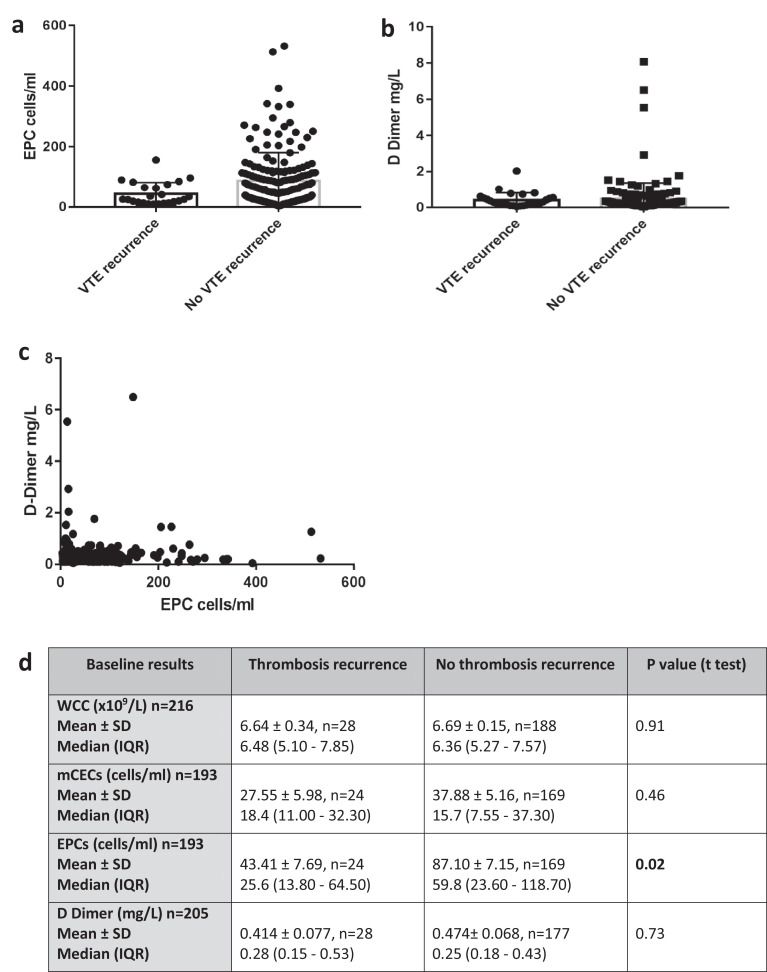
Laboratory results from peripheral blood samples taken at baseline prior to randomisation (all patients on anticoagulation at this point). Endothelial progenitor cells (EPC). Data represented is mean ± SD (a), d-dimer (b) and relationship of D Dimer and EPC results (c). Table (d) also includes results for WCC=White cell count, mCECs= Mature circulating endothelial cells.

**Fig. 3:**
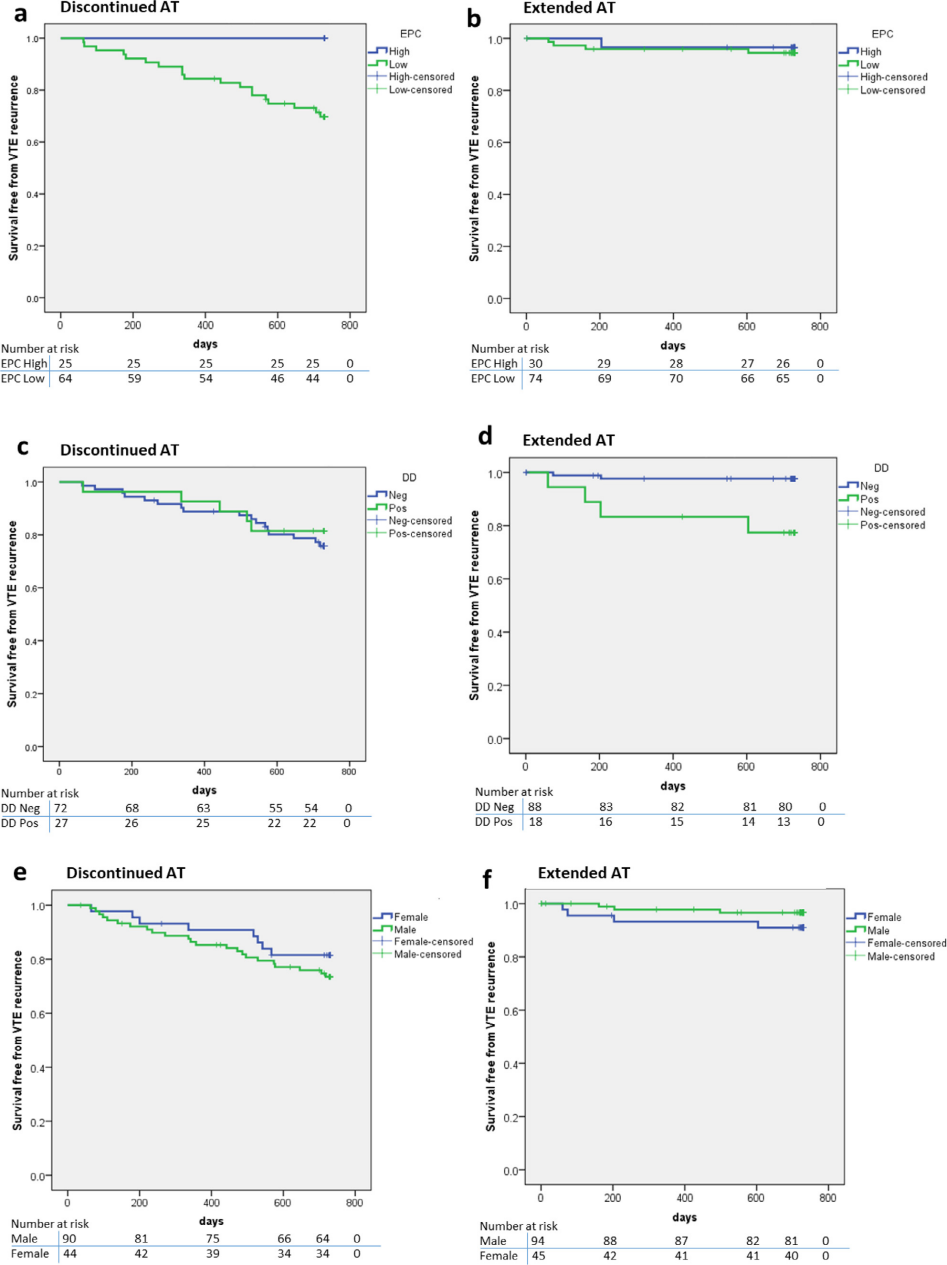
Comparison of survival free from VTE recurrence. EPC high versus low in discontinued AT group (a) and extended AT group (b). d-dimer positive versus negative in discontinued AT group (c) and extended AT group (d), Males vs females in observation group (e) and warfarin continuation group (f).

**Fig. 4:**
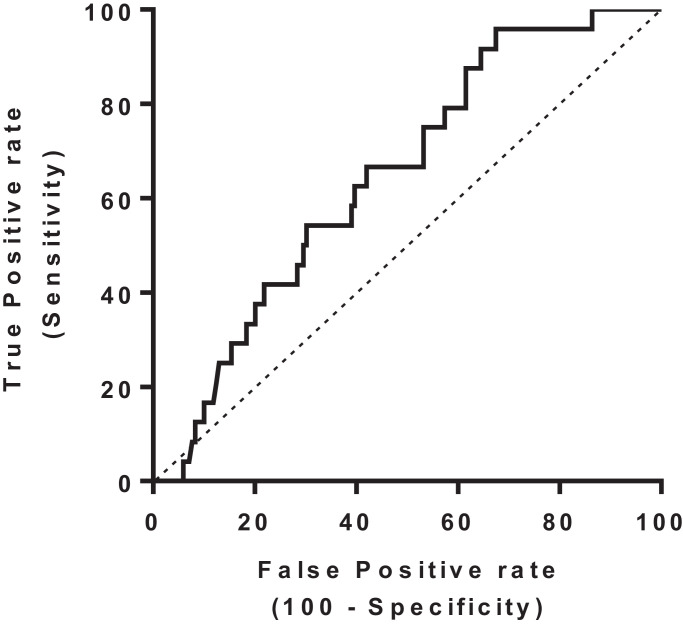
Area under receiver operating characteristic (AUROC) curve for EPC count (cells/ml) in predicting VTE recurrence. AUROC is 0.64 (95% CI 0.54 to 0.75, *p* = 0.02). Dashed line represents chance performance (0.50). An EPC threshold of <100 cells/ml gives a sensitivity of 95.83% and specificity of 30.77% to predict VTE recurrence.

At the baseline appointment, prior to randomisation, blood samples from 193 patients (all on warfarin) were collected for Endothelial cell quantitation and of these 24 (12%) went on to develop recurrent VTE (19 in the observation only group and 5 in the warfarin continuation group). Patients who went on to develop VTE recurrence had a significantly lower level of endothelial progenitor cells at baseline than those who didn't (Fig. 2a and 2d, 43.41 cells/ml ± 7.69 vs 87.1 ± 7.15 cells/ml, *p* = 0.02 by *t*-test). The survival free from VTE recurrence (Fig. 3) for patients with EPCs ≥ 100 cells/ml (*n* = 55) was significantly improved compared to those with EPCs <100 cells/ml, *n* = 138 (adjusted hazard ratio of 0.10, 95% CI: (0.01- 0.73), *p* = 0.023 based on the Cox regression analysis. Table 2). The area under receiver operating characteristic (AUROC) curve for EPC count (cells/ml) in predicting VTE recurrence is shown in Fig. 4. AUROC is 0.64 (95% CI 0.54 to 0.75, *p* = 0.02). Dashed line represents chance performance (0.50). An EPC threshold of <100 cells/ml gives a sensitivity of 95.83% and specificity of 30.77% to predict VTE recurrence.

### 4.4 Other baseline blood results

There was no significant difference at baseline in d-dimers (*n* = 205), White cell count (*n* = 216) and mCECs (*n* = 193) between patients who went on to develop VTE recurrence versus those who didn't (Fig. 2b and 2d). High d-dimer results tended to be associated with low EPC quantitation and high EPC levels associated with low d-dimers (Fig. 2C). Patients with a positive d-dimer (≥0.5 mg/L) at baseline (*n* = 45) did not have a significant reduction in survival free from VTE recurrence compared to those with a negative d-dimer (*n* = 160) (adjusted hazard ratio of 1.41 (95% CI 0.63–3.14 *p* = 0.400 by Cox regression)) (Table 2, Fig. 3).

## 5 Discussion

This is the first report of EPC measurement in a VTE clinical trial. In particular, this is the first time EPC quantitation has been evaluated as a laboratory biomarker for prediction of VTE recurrence risk. Focus to-date has been on identifying a useful plasma marker rather than a cellular marker for VTE. This is perhaps surprising given the integral role of the vascular endothelium in haemostasis.

The traditional view that fibrinolysis is the predominant mechanism of clot resolution needs challenging. Thrombus resolution is a complex process involving organisation, formation of neovascular channels, neovascularization, fibrinolysis and inflammation within the thrombus [28]. There is accumulating evidence that EPCs are essential components of the natural “repair” process following thrombosis [21,22,28,29]. Previous studies have demonstrated that EPCs can accelerate recanalization of thrombus by expression or secretion of vasoactive and angiogenic factors, restoring lost or damaged endothelium, enhancing neovascularisation, and promoting vein recanalization. EPCs have a fundamental role in the restoration of the structural and functional integrity of the endothelial monolayer which is critical for the prevention of thrombosis and limitation of thrombus propagation. There is a growing literature on the important role and value of EPCs in arterial disease. However, although supportive, the data in VTE is limited to animal studies and small observational human studies. We hypothesised that following an unprovoked VTE, the level of EPCs circulating in the peripheral blood may be representative of the patient's ability to repair/protect the venous circulation and prevent thrombosis recurrence by maintaining endothelial integrity. If confirmed, this may have valuable prognostic information that could aid clinical decision making and potentially be a target for future VTE treatment and secondary prevention strategies.

Current clinical management of a first episode of VTE could be enhanced by identifying patients who can safely discontinue anticoagulant therapy after a minimum of 3 months anticoagulation. There is an unmet clinical need for a laboratory biomarker to define this patient group. To-date numerous clinical studies have evaluated d-dimer assays as a biomarker for VTE recurrence. The majority of these tested d-dimers after discontinuation of anticoagulation for 1 month which is logistically complex and potentially harmful, risking recurrence while awaiting testing. In addition, a negative d-dimer does not reliably define a group at low risk of VTE recurrence [10,30]. The HERDOO2 rule has recently been prospectively validated. This rule states that women with none or one of the specific criteria are at sufficiently low risk of VTE recurrence to safely discontinue anticoagulation after short term treatment (HERDOO2 criteria: Hyperpigmentation, Edema, Redness in either leg, d-dimer level ≥ 250 µg/L whilst on anticoagulation, Obesity with a BMI ≥30 or older age ≥ 65). In the cohort evaluated by Roger et al., 51.3% of women (*n* = 631 of 1213) were classified as low risk and of these, the risk of recurrence over 1 year follow up was 3% for patients who discontinued anticoagulation. In contrast, patients classified as high risk had a recurrence risk of 8.1% [31]. This rule has some advantages compared to other scores (such as DASH [6]) as it incorporates d-dimer testing when patients are still on anticoagulation, although it remains unclear how predictive d-dimers are when taken on anticoagulation when not incorporated within the HERDOO2 score. In addition, if this score is used all men and about half of women will continue indefinite duration anticoagulation and it would be valuable if a biomarker could identify additional patients who could safely discontinue anticoagulation.

In the ExACT study, d-dimer testing whilst on anticoagulation was not predictive of VTE recurrence. Previous studies have demonstrated that only a small proportion of anticoagulated patients have a positive d-dimer result, highlighting the reduced sensitivity of this assay in this context [30]. However, even those patients remaining negative after anticoagulant discontinuation, continue to have a significant long-term risk of recurrence (e.g. males 7.5% annual recurrence risk) [10]. Consistent with this, in the ExACT study, less than one quarter of patients had a positive D Dimer (quantitative laboratory testing) whilst on anticoagulation (22%, *n* = 45/205). Interestingly, a smaller proportion of patients had a positive d-dimer with point of care testing in ExACT (4.4%, *n* = 12/273). [24] Differences in positive d-dimer reporting between clinical studies are likely to reflect the variation in different qualitative and quantitative assays used and problems with laboratory standardisation. It remains controversial which d-dimer assay and what threshold is best in this context, whether single or serial testing is better and how results should influence clinical management. In the ExACT study, VTE recurrence was common in patients with a negative d-dimer who stopped anticoagulation and occurred in 23.6% of patients over 2 years (17 out of 72 patients, Table 3). Therefore, isolated measurement of d-dimer on anticoagulated patients is unlikely to be helpful to inform which patients can safely stop anticoagulation after limited duration treatment.

Contrary to previous studies, sex was not predictive of recurrence in the ExACT study, but interpretation of this is limited by patient numbers (only 1/3 female) and infrequent events in patients who continued anticoagulation. Although, the VTE recurrence event rate was higher in males who stopped anticoagulation (26% over 2 years, Table 3), this was not a statistically significant difference and women who stopped anticoagulation were still at high risk of recurrence (18% over 2 years).

In contrast, patients with a higher level of circulating EPCs were significantly less likely to develop VTE recurrence. Remarkably, only one patient with a high level of EPCs developed recurrence and this was a patient randomised to continue warfarin. Therefore, it is possible that the elevated level of EPCs represents an effective repair response to the original event and is protective against recurrence. Consistent with this, elevated levels of EPC were associated with low levels of D Dimers and visa versa (Fig. 2c).

Although our results show an association of EPC numbers and VTE recurrence, further work would be needed to confirm these cells have a mechanistic role to prevent recurrence. If EPCs are directly involved in repair and recanalization following VTE, enhancing their numbers could have therapeutic implications. It is known that thrombolysis (either systemic or catheter directed) administered early after VTE event, is associated with faster vein recanalization which facilitates valve integrity preservation and alleviates venous obstruction [32]. However, catheter directed thrombolysis has not consistently demonstrated a long-term benefit in terms of reduced post thrombotic syndrome [33]. Thrombolysis also only works if used early after an acute VTE with very limited efficacy later on, once thrombosis is established. In addition, there is a significant risk of serious bleeding and for most patients the risks of thrombolysis outweigh the benefits. It may be that therapeutic strategies to increase recruitment of endothelial progenitor cells could promote effective vein recanalization, even when thrombosis is established and improve clinical outcomes without the bleeding consequences of thrombolysis. In addition, treatment strategies that increase circulating EPC levels may be a new therapeutic option to reduce the risk of recurrent VTE. Of interest, statins have previously been reported to increase EPC levels and it is possible this is the mechanism to explain the reduction in VTE recurrence risk reported with their use [18,34].

The strengths of the ExACT study include the relatively large sample size (for testing a novel biomarker) and significant length of clinical follow up of 2 years. A limitation of this study is that only half of patients were randomised to stop anticoagulation and recurrence in the other half (patients who continued anticoagulation) was very low (*n* = 7 of 141, 5% over 2 years). A further limitation is that 45 participants (34% of 134) in the discontinued anticoagulation group and 35 participants (25% of 139) in the extended anticoagulation group did not have blood samples taken for EPC quantitation. Of the 80 participants without EPC measurements, 14 had VTE recurrence over 2 years (12 were in the observation only group and 2 in the warfarin continuation group). In addition, all patients in ExACT were on warfarin when blood samples were taken for EPC quantitation and it is possible that results from patients on DOACs would not be equivalent. Another limitation is that the EPC cut off for “high” vs “low” was determined post hoc. Therefore, further studies are needed to prospectively validate this biomarker and this threshold in a larger patient cohort treated with DOACs to confirm the clinical utility of this assay. Further studies are also needed to compare EPC levels with other clinical outcomes such as post thrombotic syndrome or chronic thromboembolic pulmonary hypertension.

In summary, in this relatively large cohort with 2 years follow up, EPC quantitation shows promise as a novel biomarker to predict VTE recurrence risk. The additional benefit of this relatively straightforward, inexpensive assay is the ability to test patients who are still on anticoagulation, which overcomes the logistical complexities of d-dimer testing. Flow cytometry cellular quantitation assays are widely available and used for many diagnostic and prognostic indications (e.g. in haemato-oncology). This assay would be expected to have similar standardisation procedures, availability and limitations (in particular the need to analyse samples while cells are still viable). The high sensitivity (95.83%, Fig. 4) of an EPC level <100 cells/ml to VTE recurrence, suggests that patients with EPC levels above this threshold have a very low VTE recurrence risk. If confirmed, EPC quantitation could add valuable information to clinical decision making at 3 months by the identification of a truly “low risk group” who can safely stop anticoagulation. The ability to stop anticoagulation after a defined treatment period in patients who no longer need it has advantages in terms of bleeding risk, financial and resource cost savings and patient convenience. Consistent with the published literature, these results also support a mechanistic importance of EPCs following VTE with potential for a new avenue of therapeutic strategies.

## Declaration of Competing Interest

None of the authors have conflicts of interest relevant to this manuscript. Dr Bradbury reports personal fees from BMS Pfizer, other from Bayer, other from Amgen, personal fees from Novartis, personal fees from Janssen, personal fees and other from Ablynx. Dr Buckley, Dr Sun, Dr Rose and Prof Fitzmaurice have nothing to disclose.

## Appendix Supplementary materials

Image, application 1

Image, application 2
