# Supplementary material for: Patients with high levels of circulating endothelial progenitor cells (EPC) following at least three months of anticoagulation for unprovoked venous thromboembolism (VTE) are at low risk of recurrent VTE—Results from the ExACT randomised controlled trial
Source: eClinicalMedicine. 2019 Nov 27;17:100218. doi: 10.1016/j.eclinm.2019.11.011 (PMC6933150; doi:10.1016/j.eclinm.2019.11.011)
Supplement: Supplementary file 1 [file mmc1.docx]

Figure 1 Patient Flow following randomisation

N=134

Data available for analysis of primary outcome (time to the first recurrent venous thromboembolism since randomisation up to 24 months follow up)

Excluded from analysis (N=6)

Consent to use data withdrawn (N=4)

Participant with antiphospholipid syndrome (N=0)

Participant with protein C and/or protein S or antithrombin deficiency (N=2)

**Randomised**

N=281

**Discontinued anticoagulation therapy (AT)**

N=140

**Extended anticoagulation therapy (AT)**

N=141

*Withdrawn (N=6)**

*Withdrawn (N=2)***

N=139***

Data available for analysis of primary outcome (time to the first recurrent venous thromboembolism since randomisation up to 24 months follow up)

Excluded from analysis (N=2)

Consent to use data withdrawn (N=1)

Participant with antiphospholipid syndrome (N=1)

Participant with protein C and/or protein S or antithrombin deficiency (N=0)

* Includes 4 participants who withdrew consent to use their data

** Includes 1 participant who withdrew consent to use their data

*** Includes 2 patients receiving rivaroxaban therapy

Table 1- Baseline Characteristics

| **Characteristic** | | **Discontinued AT**  N=134 | **Extended AT**  N=139 | **Total**  N=273 |
| --- | --- | --- | --- | --- |
| **Age at time of randomisation** | |  | |  |
|  | Mean (SD) | 63.3 (12.7) | 62.2 (13.0) | 62.7 (12.8) |
|  | Median (IQR) | 64.5 (55.6-74.0) | 64.4 (53.3-72.4) | 64.4 (54.4-72.7) |
| **Sex, n (%)** | |  |  |  |
|  | Female | 44 (32.8) | 45 (32.4) | 89 (32.6) |
|  | Male | 90 (67.2) | 94 (67.6) | 184 (67.4) |
| **Diagnosis (DVT/PE)^1^, n (%)** | |  |  |  |
|  | Unprovoked DVT | 69 (51.5) | 70 (50.4) | 139 (50.9) |
|  | Unprovoked PE | 65 (48.5) | 69 (49.6) | 134 (49.1) |
| **Ethnicity, n (%)** | |  |  |  |
|  | White | 131 (97.8) | 131 (94.2) | 262 (96.0) |
|  | Mixed | 1 (0.8) | 0 (0.0) | 1 (0.4) |
|  | Asian or Asian British | 0 (0.0) | 3 (2.2) | 3 (1.1) |
|  | Black or Black British | 2 (1.5) | 5 (3.6) | 7 (2.6) |
|  | Other ethnic groups | 0 (0.0) | 0 (0.0) | 0 (0.0) |
| **Smoking status, n (%)** | |  |  |  |
|  | Non-smoker | 63 (47.0) | 60 (43.2) | 123 (45.1) |
|  | Ex-smoker | 48 (35.8) | 60 (43.2) | 108 (39.6) |
|  | Current smoker | 19 (14.2) | 18 (13.0) | 37 (13.6) |
|  | Smokes occasionally | 4 (3.0) | 1 (0.7) | 5 (1.8) |
| **Alcohol consumption, n (%)** | |  | |  |
|  | No | 44 (32.8) | 51 (36.7) | 95 (34.8) |
|  | Yes | 90 (67.2) | 88 (63.3) | 178 (65.2) |
| **BMI classification, n (%)** | |  |  |  |
|  | Underweight (<18.5) | 2 (1.5) | 0 (0.0) | 2 (0.7) |
|  | Normal range (18.5-24.99) | 47 (35.1) | 47 (33.8) | 94 (34.4) |
|  | Overweight (25-29.99) | 51 (38.1) | 53 (38.1) | 104 (38.1) |
|  | Obese (≥30) | 33 (24.6) | 37 (26.6) | 70 (25.6) |
|  | Missing | 1 (0.8) | 2 (1.4) | 3 (1.1) |
| **Family history of VTE, n (%)** | |  |  |  |
|  | No | 102 (76.1) | 102 (73.4) | 204 (74.7) |
|  | Yes | 32 (23.9) | 37 (26.6) | 69 (25.3) |
| **Previous medical history** | |  |  |  |
| **Stroke, n (%)** | |  |  |  |
|  | No | 130 (97.0) | 136 (97.8) | 266 (97.4) |
|  | Yes | 4 (3.0) | 3 (2.2) | 7 (2.6) |
| **Transient Ischaemic Attack (TIA), n (%)** | |  |  |  |
|  | No | 129 (96.3) | 138 (99.3) | 267 (97.8) |
|  | Yes | 5 (3.7) | 1 (0.7) | 6 (2.2) |
| **Angina, n (%)** | |  |  |  |
|  | No | 129 (96.3) | 136 (97.8) | 265 (97.1) |
|  | Yes | 5 (3.7) | 3 (2.2) | 8 (2.9) |
| **Myocardial Infarction (MI), n (%)** | |  |  |  |
|  | No | 133 (99.3) | 134 (96.4) | 267 (97.8) |
|  | Yes | 1 (0.8) | 5 (3.6) | 6 (2.2) |
| **Ischaemic Heart Disease (IHD), n (%)** | |  |  |  |
|  | No | 130 (97.0) | 136 (97.8) | 266 (97.4) |
|  | Yes | 4 (3.0) | 3 (2.2) | 7 (2.6) |
| **Peripheral Vascular Disease (PVD), n (%)** | |  |  |  |
|  | No | 134 (100.0) | 134 (96.4) | 268 (98.2) |
|  | Yes | 0 (0.0) | 5 (3.6) | 5 (1.8) |
| **PTS score (categorical), n (%)** | |  |  |  |
|  | No PTS | 70 (52.2) | 66 (47.5) | 136 (49.8) |
|  | Mild PTS | 42 (31.3) | 51 (36.7) | 93 (34.1) |
|  | Moderate PTS | 15 (11.2) | 18 (13.0) | 33 (12.1) |
|  | Severe PTS | 5 (3.7) | 2 (1.4) | 7 (2.6) |
|  | Missing | 2 (1.5) | 2 (1.4) | 4 (1.5) |
| **PTS score** | |  |  |  |
|  | Mean (SD) | 5.2 (4.2) | 5.1 (3.8) | 5.2 (4.0) |
|  | Median (IQR) | 4.0 (2.0-7.5) | 5.0 (2.0-8.0) | 4.0 (2.0-8.0) |
|  | Missing | 2 | 2 | 4 |
| **EQ-5D-3L** | |  |  |  |
|  | Mean (SD) | 0.8 (0.2) | 0.8 (0.3) | 0.8 (0.3) |
|  | Median (IQR) | 0.8 (0.7-1.0) | 0.8 (0.7-1.0) | 0.8 (0.7-1.0) |
|  | Missing | 0 | 4 | 4 |
| **VEINES-QOL score** | |  |  |  |
|  | Mean (SD) | 48.2 (10.7) | 49.6 (9.9) | 48.9 (10.3) |
|  | Median (IQR) | 51.1 (41.1-57.6) | 53.0 (44.6-56.7) | 52.1 (43.3-57.5) |
|  | Missing | 0 | 2 | 2 |
| **Health care utilisation due to PTS** | |  |  |  |
| **Patient receiving primary care  treatment, n (%)** | |  |  |  |
|  | No | 124 (92.5) | 128 (92.1) | 252 (92.3) |
|  | Yes | 9 (6.7) | 11 (7.9) | 20 (7.3) |
|  | Missing | 1 (0.8) | 0 (0.0) | 1 (0.4) |
| **Type of nurse patients were  seen by, n (%)** | |  |  |  |
|  | Practice | 2 (1.5) | 1 (0.7) | 3 (1.1) |
|  | District | 0 (0.0) | 0 (0.0) | 0 (0.0) |
|  | HCA | 0 (0.0) | 0 (0.0) | 0 (0.0) |
|  | None | 59 (44.0) | 70 (50.4) | 129 (47.3) |
|  | Other | 0 (0.0) | 0 (0.0) | 0 (0.0) |
|  | Missing | 73 (54.5) | 68 (48.9) | 141 (51.7) |
| **Patient receiving treatment for a leg ulcer, n (%)** | |  |  |  |
|  | No | 66 (49.3) | 71 (51.1) | 137 (50.2) |
|  | Yes | 1 (0.8) | 2 (1.4) | 3 (1.1) |
|  | Missing | 67 (50.0) | 66 (47.5) | 133 (48.7) |
| **Patient receiving secondary care treatment, n (%)** | |  |  |  |
|  | No | 131 (97.8) | 135 (97.1) | 266 (97.4) |
|  | Yes | 1 (0.8) | 4 (2.9) | 5 (1.8) |
|  | Missing | 2 (1.5) | 0 (0.0) | 2 (0.7) |
| ^1^minimisation variable | | | | |
